# The epidemiology of mescaline use: Pattern of use, motivations for consumption, and perceived consequences, benefits, and acute and enduring subjective effects

**DOI:** 10.1177/02698811211013583

**Published:** 2021-05-05

**Authors:** Malin Vedøy Uthaug, Alan K Davis, Trevor Forrest Haas, Dawn Davis, Sean B Dolan, Rafael Lancelotta, Christopher Timmermann, Johannes G Ramaekers

**Affiliations:** 1 Department of Neuropsychology and Psychopharmacology, Faculty of Psychology and Neuroscience, Maastricht University, Maastricht, The Netherlands; 2 College of Social Work, The Ohio State University, Columbus, OH, USA; 3 Center for Psychedelic and Consciousness Research, Department of Psychiatry and Behavioral Sciences, Johns Hopkins School of Medicine, Baltimore, MD, USA; 4 University of California, Davis School of Medicine, Sacramento, CA, USA; 5 Department of Natural Resources, University of Idaho, Moscow, ID, USA; 6 Behavioral Pharmacology Research Unit, Department of Psychiatry and Behavioral Sciences, Johns Hopkins University School of Medicine, Baltimore, MD, USA; 7 Habituating to Wholeness, Lakewood, CO, USA; 8 Centre for Psychedelic Research, Department of Brain Sciences, Imperial College London, London, UK

**Keywords:** Mescaline, 3,4,5-trimethoxyphenethylamine, epidemiology, survey

## Abstract

**Background:**

Mescaline is a naturally occurring psychoactive phenethylamine found in several cacti and historically used ceremonially by Indigenous and Latin American populations. Broader recognition of its possible therapeutic value in Western science began in the 1950s; however, knowledge of the safety profile of mescaline and the extent of its use remains limited. The primary aim of this study is to examine the epidemiology of mescaline use among English-speaking adults.

**Methods:**

About 452 respondents completed a web-based survey designed to assess their previous experience with mescaline (subjective effects, outcome measures, and mescaline type used).

**Results:**

Most respondents reported that they had consumed mescaline infrequently (⩽once/year), for spiritual exploration or to connect with nature (74%). A small number of respondents reported drug craving/desire (9%), whereas very few reported legal (1%), or psychological problems (1%) related to its use, and none reported seeking any medical attention. Overall, respondents rated the acute mystical-type effects as “*moderate*,” ego-dissolution and psychological insight effects as “*slight*,” and challenging effects as “*very slight*.” Most respondents reported that they used Peyote and San Pedro in their most memorable mescaline experience. Overall, the intensity of acute mescaline effects did not differ between mescaline types. About 50% of the sample reported having a psychiatric condition (i.e. depression, anxiety, etc.), and most (>67%) reported improvements in these conditions following their most memorable experience with mescaline.

**Conclusion:**

Findings indicate that the mescaline in any form may produce a psychedelic experience that is associated with the spiritual significance and improvements in the mental health with low potential for abuse.

## Introduction

Mescaline (3,4,5-trimethoxyphenethylamine) is a naturally occurring psychoactive alkaloid within the substituted phenethylamine class and is typically consumed as raw plant material, extractions from plants, or synthetic preparations (Jay, 2019). In its naturally occurring form, mescaline is the primary psychoactive compound in two cactus species: the North American *Lophophora williamsii*, also known as Peyote, and the South American *Trichocereus pachanoi*, also known as San Pedro or “huachuma” (Jay, 2019). Several other cacti species also reportedly contain mescaline, primarily within the *Trichocereus* and *Echinopsis* genera (Trout, 2005). Mescaline was first isolated from *L. williamsii* in 1896 by German chemist Arthur Heffter (Garcia-Romeu et al., 2016), and later synthesized in 1919 by Ernst Spaff (Rucker et al., 2018). Direct archaeological evidence of mescaline-containing cacti indicates that its use is among the oldest of psychoactive substances in the New World (Samorini, 2019). Evidence suggests that mescaline-containing cacti have been used ceremonially by Indigenous cultures in the USA, Mexico, and Peru for more than 7000 years (Schultes et al., 1992), despite efforts by early Spanish conquerors to eradicate the practice beginning in the 16th century (Jay, 2019). The first Western medical report of Peyote consumption was recorded in the late 19th century (Prentiss and Morgan, 1895), with the first administration of isolated mescaline reported some two decades later (Knauer and Maloney, 1913).

Pharmacologically, mescaline is a long-acting, low-potency psychedelic phenethylamine substance (Dasgupta, 2019). Mescaline, like other classic psychedelic compounds, exerts its pharmacological action primarily at 5-HT_1A_, 5-HT_2A_, and alpha-2 adrenergic receptors (Ray, 2010; Rickli et al., 2015), although the primary sensory and cognitive effects result from modulation of serotonergic 5-HT_2A_ receptors (Carstairs and Cantrell, 2010; Nichols, 2016). Mescaline exhibits very low binding affinity at dopaminergic and histaminergic receptors and does not inhibit uptake at monoamine transporters (Rickli et al., 2016). The 3,4,5-trimethoxy configuration of mescaline appears central to its psychedelic activity (Smythies et al., 1967).

Effective oral dosage of synthetic mescaline is in the 200–400 mg range, with three orders of magnitude greater than the equivalent dose of lysergic acid diethylamide (LSD) (Beyerstein, 2003; Nichols, 2004). Oral ingestion of mescaline appears to have a longer half-life compared to other classic psychedelics (i.e. 6 h), with peak effects occurring approximately 2 h after ingestion and a total duration lasting 8–12 h (Dasgupta, 2019; Nichols, 2004). Mescaline is metabolized via oxidative deamination by liver enzymes, with 87% excreted in the urine after 24 h (Dasgupta, 2019; Monte et al., 1997), and animal models show that 28%–46% of mescaline is excreted in the urine unaltered (Cochin et al., 1951).

Preclinical data have provided the understanding of the pharmacological mechanisms of action, but less is known about the acute subjective and enduring effects associated with the consumption of mescaline and whether these effects differ among synthetic mescaline or varieties of mescaline-containing cacti. Reported visual effects from dried Peyote and synthetic mescaline include spatial distortion, distortion of color, closed-eye visual hallucinations, and synesthesia (Koelle, 1958; Luke and Terhune, 2013). Some challenging psychological effects have been reported with the use of mescaline and are similar to that of acute schizophrenia and include sensory alterations, ideas of influence, paranoia, delusions, and depersonalization (Osmond and Smythies, 1952). However, both synthetic and naturally occurring mescaline also produce varying positive emotional and cognitive effects, such as pleasant feelings, euphoria, and transcendence (Klüver, 1926; Koelle, 1958; Wallace, 1959). The nausea and vomiting have more frequently been linked to Peyote ingestion than other methods of mescaline consumption (Erowid, 2009; Nolte and Zumwalt, 1999). It was suggested that this was due to its bitter taste, as well as other chemical constituents of the plant rather than just mescaline (Schultes, 1938), as volunteers who received synthetic mescaline did not report nausea or vomiting (Hermle et al., 1992). Methods for reducing emesis include mixing the plant material with fruit juices or gelatin, or by pulverizing the Peyote buttons and placing the powder into gelatin capsules (McLaughlin and JL, 1973).

As with other psychedelics, the main adverse effects of mescaline are psychological and include anxiety, panic, and disorganized behavior (Cohen, 1960). Despite these reports, adverse events associated with mescaline use are rare, as there were only 31 total single-substance exposures to mescaline reported to California Poison Control over an 11-year period (Carstairs and Cantrell, 2010). Furthermore, there is no evidence of psychological or cognitive deficits among Native American (NA) users of Peyote (Halpern et al., 2005), although only limited number of studies exist on the matter. Furthermore, these have only been done with specific population (i.e. NA groups). Like other psychedelics, mescaline is associated with a rapid-onset tolerance period lasting 3–4 days after the use, during which repeated use is associated with diminished psychological effects. Despite its potential to rapidly promote tolerance, mescaline has not been shown to produce dependence (Kapadia and Fayez, 1970).

The prevalence of use and the access and availability of mescaline varies depending on several factors. Specifically, a 2016 survey estimated that eight million people (3%) aged 12 or older in the United States (US) had used mescaline in their lifetime (Substance et al., 2016). The same study found that 5.5 million people in the US (2%) aged 12 or older had used Peyote in their lifetime, despite Peyote’s classification as a Schedule I substance by the Drug Enforcement Agency (DEA) (2019). However, the passage of the American Indian Religious Freedom Act Amendments of 1994 (AIRFA), which protected the religious use of Peyote for enrolled members of Federally recognized Native American Tribes and members of the Native American Church (NAC), was associated with an increase of Peyote use prevalence of <10% among Native Americans (NAs) (Prue, 2014).

Today, members of the NAC report using Peyote anywhere from once per year to two to three times per week (Dasgupta, 2019). Though the AIRFA essentially decriminalized Peyote use for NAs with the 1994 amendments, the city of Oakland, CA passed a resolution, which decriminalized all “entheogenic plant medicines” including mescaline-containing cacti in June 2019 (Epstein, 2019), which may lead to an exponentially increased use by Indigenous peoples in the future. Decriminalization resolutions, which include Peyote, may contribute significantly to the extinction of the Peyote cacti in the wild. Similarly, the Republic of Peru, South America has enacted legislation protecting traditional use of Indigenous plant medicines, such as San Pedro (Dunnell, 2018).

Anecdotal Internet reports from the US describe the mescaline use for recreational, spiritual, and therapeutic purposes (Erowid, 2011). Recognized for its possible psychotherapeutic value in the 1930s, mescaline was first used therapeutically in the 1950s to help patients access repressed memories, gain insight into emotional issues, and explore ego defense mechanisms (e.g. regression, increased awareness of acute anxiety, and somatization of affect) (Cattell, 1954; Frederking, 1955; Guttmann, 1936). NA participants in Peyote ceremonies commonly experienced reductions in chronic anxiety, heightened community satisfaction, and increased sense of personal worth (Wallace, 1959). Within the NAC, Peyote has been used to treat chronic alcoholism within ethnically oriented residential treatment programs (Albaugh and Anderson, 1974). In Western communities, it has been suggested that mescaline may play a role in the treatment of obsessive compulsive disorder (OCD) and personality disorders (Delgado and Moreno, 1998; Hartogsohn, 2017). Anonymous Internet posts by recreational users describe mescaline as a means of attaining spiritual transformation, gratitude, compassion, and interconnectedness with the universe (Erowid, 2011, 2012). Although previous research suggests beneficial effects of mescaline, it is currently not approved as a medicine by any health authority, and the benefit/risk ratio of mescaline is presently unknown due to lack of rigorous clinical research.

The pharmacology of mescaline has been assessed in animal models (Bevan et al., 1974; Darvesh and Gudelsky, 2003; Kyzar et al., 2012; Nichols, 2004), and the subjective effects have been reported in numerous case studies (Frederking, 1955; Halpern, 1961; Klüver, 1926; Osmond and Smythies, 1952). Although there are reports of mescaline use on Internet sites (e.g. Erowid, BlueLight, Drugs-Forum, etc.) and among the Peyote-ingesting members of the NAC (Dasgupta, 2019; Jay, 2019; Prue, 2014), no epidemiological studies examining the patterns of use, subjective effects, motivations for use, or potential medical and psychological harms/benefits of consuming mescaline in the general population exist.

The relative absence of information about the scope of mescaline use limits understanding of the safety profile of this substance, which is needed to inform the design of future studies with this compound. Therefore, the primary aim of this study is to examine the epidemiology of mescaline use (patterns and motivation for use, subjective effects, and potential medical and psychological harms/benefits as a result of consumption) among English-speaking adults who have consumed mescaline at least once in their lifetime. As a secondary aim, we examined whether there were changes in medical and psychological functioning following mescaline use. The final aim involved examining differences in the subjective effects and the patterns and motivations of use as a function of the type of mescaline consumed (i.e. synthetic, extracted, Peyote, or San Pedro).

## Methods

### Procedure

From January 2019 to October 2019, we posted written recruitment advertisements on multiple Internet platforms (at Erowid.org, Facebook.com, etc.). Authors also shared links to the survey via personal networks. All advertisements contained the information regarding the purpose of the study and the approximate time required to complete the survey (45–60 min), and that participating in the survey was anonymous. Upon clicking any of our advertisements potential respondents were sent to a secured survey site (hosted by Qualtrics) where they viewed the informed consent document that repeated the purpose of the study and described the following eligibility criteria: at least 18 years of age, able to read and understand English, and having used mescaline at least once in their lifetime. All study procedures were approved by the Local Standing Ethical Committee at Maastricht University in the Netherlands.

### Measures

#### Mescaline survey

The survey was introduced with a description of the various types of mescaline (i.e. synthetic, San Pedro, Peyote, and extracted) of interest. During the first portion of the survey, respondents were asked to answer a question in the context of the most memorable experience with one of these of these mescaline types. That question was phrased in the following way: “Take a few moments now to recollect and bring to mind your Mescaline experience. If you have had multiple such experiences, please answer the following with respect to the MOST MEMORABLE one. Please briefly describe this Mescaline experience in the box below.” The participant was asked to answer the following survey items with this one experience in mind. The survey also included items on the administration route (i.e. swallowed, snorted, smoked/vaporized, injected, sublingual, and rectal), how the mescaline was obtained (i.e. online, purchased online, licensed distributor provided at a ceremony, etc.), age at the time of the mescaline experience, location of their experience, whether there were other people present when they consumed mescaline, and if so, how many other people were also using mescaline. Additionally, the survey asked about the main reason for consuming mescaline, how participants would characterize the quantity of the dose(s) (low, moderate, etc.), how many times they ingested mescaline during the session, the duration of effects, how they prepared for their experience, and how many times they used mescaline before and after this session. Moreover, respondents were asked about their medical/psychological history (i.e. had a medical/psychological condition in the past), and whether their condition had improved, remained the same, or worsened following mescaline use.

The second part of the survey included questions about “lifetime use of mescaline.” Respondents were asked what types of mescaline had ever been ingested in their lifetime, age at first use, administration route, frequency, reason, and location of use. Furthermore, this part of the survey also included variables assessing several aspects of abuse potential, such as frequency of repeated consumption in the same session, craving/desire for mescaline, possible consequences they may have experienced related to mescaline use (e.g. a visit to the Emergency Department or Urgent care), and finally whether they ever attempted to quit, reduce, or increase their consumption. This section also asked about dose, source, and preparation, as well as how many people were present during the session and whether they were also consuming mescaline. In addition, respondents were asked about psychological or spiritual applications of the use of mescaline, and to estimate the use of other (psychoactive) substances they had used during their lifetime. The full survey is available upon request from the corresponding author.

#### Subjective acute and enduring effects

##### Psychological insight questionnaire

The psychological insight questionnaire (PIQ) consists of 23 items that assess the intensity with which respondents experienced a broad range of insights (e.g. gained an awareness into emotions, behaviors, beliefs, memories, or relationships) after taking a classic psychedelic (Davis et al., 2020a, 2020b). Respondents were asked to reflect on their most memorable experience with mescaline and to rate the degree to which they, at any time during that session, experienced a broad range of insights on a 5-point scale from 0 = “None; not at all” to 5 = “Extremely.” The internal consistency of the total scale in the current sample was excellent (Cronbach’s alpha = 0.95).

##### Acute mystical experiences

The acute mystical experiences (MEQ-30) is a 30-item self-report scale of mystical experience (Barrett et al., 2015; MacLean et al., 2011). Respondents were asked to reflect on their most memorable experience with mescaline and to describe the intensity with which they experienced each mystical effect. The scale has four factors: (1) mystical, (2) positive mood, (3) transcendence of time/space, and (4) ineffability as described in the study by Barrett et al. (2015). Each item was rated on a five-point scale from 0 = “None; not at all” to 5 = “Extreme.” Higher scores indicate stronger mystical experiences. A “complete mystical experience” is counted when ⩾60% of the maximum possible score is endorsed on all four MEQ subscales. The internal consistency of the total scale in the current sample was excellent (Cronbach’s alpha = 0.96).

##### Challenging experience questionnaire

The challenging experience questionnaire (CEQ) is a 26-item self-report measure that assesses the intensity of challenging experiences after taking a psychedelic (Barrett et al., 2016). Respondents were asked to reflect on their most memorable experience with mescaline and to describe the intensity and challenge with which they experienced each psychological or physical experience using a five-point scale from 0 = “None; not at all” to 5 = “Extreme.” Previous research (Barrett et al., 2016) has found that the measure produces seven subscales: (1) fear, (2) grief, (3) physical distress, (4) sanity, (5) isolation, (6) death, and (7) paranoia. We also calculated a CEQ total score to rate the overall intensity of challenging experiences. Internal consistency of the total scale in the current sample was excellent (Cronbach’s alpha = 0.92).

##### Ego-dissolution inventory

The ego-dissolution inventory (EDI) is an eight-item self-report measure that assesses the level of ego-dissolution after ingesting a hallucinogen (Nour et al., 2017). We altered the phrasing of the instructions and the respondents were asked to reflect on their most memorable experience with mescaline and to describe the intensity with ego-dissolution on an altered scale that ranged from 0 = “None; not at all” to 5 = “Extreme,” instead of the original sliding scale from 0% to 100%. The internal consistency of the total scale in the current sample was excellent (Cronbach’s alpha = 0.91).

##### Persisting effects questionnaire

The persisting effects questionnaire (PEQ) assesses desirable and undesirable enduring effects of a hallucinogenic experience (Griffiths et al., 2006, 2011; Johnson et al., 2014). Respondents were asked to reflect on their most memorable experience with mescaline and to rate the experience degree: personally meaningful, spiritually significant, psychologically challenging, and psychologically insightful (on a scale from 0 = “No more than routine, everyday experiences” to 7 = “The single most meaningful experience of my life”). Moreover, respondents were asked whether their experience with mescaline had led to any enduring changes in their current sense of personal well-being or life satisfaction, life’s purpose, life’s meaning, social relationships as a whole, attitudes about life, attitudes about self, relationship to nature, behavioral changes, spirituality, attitudes about death, and views regarding the true nature of reality and the universe (on a scale from 3 = “Strong positive change that I consider desirable” to −3 = “Strong negative change that I consider undesirable”). As there is no total scale for this measure, these are all looked at as individual items.

### Demographics

*These questions assessed a variety of demographic variables* (e.g. age, gender, ethnicity, sexual orientation, country of residence, employment status, highest level of education, and relationship status).

### Data analysis

The first step of the analysis consisted of calculating frequency counts and analyzing descriptive demographic characteristics, patterns of mescaline use, subjective mystical, challenging, ego-dissolution, psychological insight effects, and psychological harms/benefits variables related to the type of mescaline that respondents reported they had the most memorable experience with (i.e. synthetic, San Pedro, Peyote, and extracted). Later, using a series of chi-square tests (with follow-up post-hoc *z*-tests examinations with the Bonferroni corrections), we examined differences in demographic characteristics (i.e. gender, race, ethnicity, country of residence, sexual orientation, employment status, education, and marital status), mescaline use variables (i.e. subjective dose level, duration of experience, location of experience, source of mescaline, and reasons for use), and other background variables (e.g. mental health conditions) as a function of the type of mescaline that respondents reported they had the most memorable experience with (i.e. synthetic, San Pedro, Peyote, and extracted). Furthermore, using a series of one-way ANOVA (with follow-up post-hoc tests with the Bonferroni corrections) tests, we examined differences in demographic characteristics (i.e. age of respondent), mescaline use variables (i.e. number of doses consumed and number of mescaline experiences), subjective acute effects (e.g. mystical, challenging, ego-dissolution, insight, personal meaning, etc.), and enduring effects (e.g. persisting changes in mood, attitudes, behavior, etc.) as a function of the type of mescaline that respondents reported they had the most memorable experience with (i.e. synthetic, San Pedro, Peyote, and extracted). Analyses were conducted using the IBM SPSS Statistics v.25 and v.26 (IBM Corp., Armonk, NY, USA).

## Results

### Respondent characteristics

During recruitment (January 2019 to October 2019), a total of 2025 people clicked one of the recruitment ads and were presented with the information about the research study. Of these individuals, 788 consented to participate and began filling out the survey; however, only 477 completed all the main study questionnaires (described above) and reported that their responses were “valid.” Of these respondents, we excluded 22 because they did not know or were unable to identify what form of mescaline, they had the most memorable experience with (i.e. synthetic, San Pedro, Peyote, and extracted), and therefore they would have been excluded in analyses of subgroup differences. Of the remaining 455, an additional three were excluded because they reported being under the age of 18 (exclusion criteria). Thus, the final sample was comprised of 452 respondents.

As shown in Table 1, most survey respondents were Caucasian (83%), male (76%), and heterosexual (82%), with a mean age of 38 years (standard deviation (SD) = 14.4) and resided in North America (60%). Comparing demographic characteristics across the four mescaline subgroups revealed that respondents in the San Pedro group were younger (mean (M) = 35.6; SD = 12.6) than those in the Peyote (M = 47.0; SD = 16.7) or synthetic (M = 42.9; SD = 16.4) subgroups. Furthermore, there were larger proportions of respondents from North America in the Peyote subgroup (75%) compared to the San Pedro subgroup (53%). Lastly, larger proportions of respondents in the Peyote subgroup (37%) reported having an advanced degree compared to those in the San Pedro subgroup (23%). See Table 1 for further characteristics of this sample including *t*-tests, chi-square, and effect size data.

**Table 1.** Demographic characteristics of total sample and each subsample based each of the “most memorable” mescaline experience subgroups.

Characteristic	Total sample	Synthetic	San Pedro	Peyote	Extracted	χ^2^ or *F*	Post-hoc	Effect size
	*N* = 452	*N* = 70	*N* = 218	*N* = 98	*N* = 66			
	M (SD) or %	M (SD) or %	M (SD) or %	M (SD) or %	M (SD) or %			
Race						3.479	NS	NS
White/Caucasian	83%	89%	83%	79%	86%			
Age	38.0 (14.4)	42.9 (16.4)	35.6 (12.6)	47.0 (16.7)	38.0 (14.4)	14.656**	S = P > SP & E < P	0.10
Gender (*n* = 446)						8.051*		0.13
Male	76%	88%	75%	69%	77%		P < S	
Residence (*n* = 447)						27.699**		0.25
North America	60%	66%	53%	75%	58%		P > SP	
Europe	20%	27%	20%	17%	20%		SP < P	
Other	20%	7%	28%	8%	23%		S = P < SP	
Hispanic ethnicity						7.249	NS	NS
Hispanic	8%	3%	9%	13%	5%			
Sexual orientation (*n* = 442)						2.621	NS	NS
Heterosexual	82%	80%	79%	86%	86%			
Employment						2.669	NS	NS
Employed	68%	64%	69%	63%	74%			
Highest education level						17.618*		0.20
High school or less	12%	7%	12%	10%	20%			
Some college/2-year degree	36%	31%	37%	35%	38%			
Bachelor’s degree	26%	36%	29%	18%	20%			
Advanced degree	26%	26%	23%	37%	23%		P > SP	
Marital status						8.232*		
Married/Partnered	57%	57%	51%	62%	68%		NS	

Variables may not equal 100% due to rounding error. *N* is varied due to participants choosing “prefer not to answer” on specific items.

E: extracted; NS: non-significant; P: Peyote; S: synthetic; SP: San Pedro.

* *p* < 0.05. **p < 0.001.

### The epidemiology of mescaline use

As shown in Table 2, most respondents (66%) had consumed San Pedro in their lifetime, with smaller proportions of respondents having ingested Peyote (36%) and synthetic mescaline (31%). Overall, respondents reported that they had the most experience with San Pedro (45%). Almost all respondents reported that they had consumed mescaline through oral ingestion (97%), very small proportions reported ingesting by snorting (1%) or via sublingual administration (2%), and most (67%) reported that they last consumed mescaline at least 6 months, prior to survey participation. Most respondents (65%) reported that their first use of mescaline occurred between the ages of 18 and 30, and almost one-half (46%) reported that they have used mescaline a total of one to three times in their lifetime, with approximately one-quarter (23%) reporting lifetime use of more than 11 occasions. In terms of the frequency of mescaline consumption, most respondents (60%) reported that they generally used mescaline less than once per year, with smaller proportions reporting mescaline use more than once per year but less than monthly (23%), and notably small proportions reporting monthly use (3%). Almost all respondents reported being motivated to consume mescaline as a means to explore their spirituality or connect with nature (74%), and most believed that mescaline had potential applications for personal growth (90%), spiritual growth (87%), psychotherapeutic work (81%), enhancing creative abilities (76%), and enhancing cognitive abilities (61%). One-half of respondents reported that they primarily consumed mescaline outdoors (47%), and three-quarters reported that they consumed mescaline without oversight of another person (i.e. self-administering the mescaline) (78%).

**Table 2.** Epidemiology of mescaline use in the total sample (*N* = 452).

Characteristic/variable	Total sample
	*N* = 452
	M (SD) or %
Types of mescaline ever ingested (could select more than one)	
Synthetic	31%
San Pedro	66%
Peyote	36%
Extracted	27%
Partial extraction	23%
Other columnar cacti	12%
Unsure	2%
Types of mescaline you have the most experience with	
Synthetic	17%
San Pedro	48%
Peyote	18%
Extracted	8%
Partial extraction	7%
Other columnar cacti	1%
Unsure	1%
Age first ingested mescaline (*n* = 451) (years)	
Younger than 18	13%
18–20	24%
21–23	17%
24–26	14%
27–30	10%
31–40	11%
41–50	8%
Older than 50	4%
Route of administration	
Swallowed	97%
Snorted	1%
Smoked/vaporized	<1%
Injected	<1%
Sublingual	2%
Rectal	<1%
Number of lifetime uses of mescaline (*n* = 448)	
1	22%
2	10%
3	14%
4–10	31%
11–20	9%
21 or more	14%
Frequency of use during past 5 years	
More than once a month	3%
Less than once each month	3%
Less than once per month but more than once per year	23%
About once per year	10%
Less than once per year	60%
Main reason for consuming mescaline	
Explore/connect	74%
Treatment/healing	8%
Other (e.g. recreation)	18%
Location	
Indoors	43%
Outdoors	47%
Other (e.g. festival)	10%
Who administered the mescaline (could select more than one)	
Self-administration	78%
Shamanic practitioner	23%
Other (e.g. peer and friend)	8%
How often do you take more than one dose in a session	
Never	55%
Sometimes	29%
Frequently	6%
Always	10%
Average number of doses consumed in a session (*N* = 447)	
1	69%
2	15%
3	9%
4 or more	7%
How often consumed mescaline at the same time as other substances	
Never	44%
Once or twice	32%
More than a few times	16%
Always or almost always	8%
Ever experienced craving for mescaline	
Yes	9%
How far in advance do you typically plan before a mescaline session	
No planning	18%
A few days	26%
A week	26%
One month	24%
More than size months	6%
Source of mescaline	
Purchased	42%
Gifted	14%
Grew/found	26%
Other	18%
Changes in frequency of use in the past year	
Decreased	51%
Stayed the same	39%
Increased	10%
How many times tried to reduce consumption (*N* = 448)	
0	97%
1 or more	3%
How many times tried to quit consuming (*N* = 445)	
0	97%
1	3%
How many times tried to increase consumption (*N* = 444)	
0	60%
1	19%
2	5%
3	4%
4 or more	13%
When was the last time consumed	
Within the past month	14%
Between 1 and 6 months ago	19%
Between 6 and 12 months ago	12%
More than 1 year ago	55%
Ever arrested or in trouble with the law because of mescaline	
Yes	1%
Every been in therapy/psychiatric services as a result of mescaline	
Yes	<1%
Ever sought medical treatment as a result of mescaline	
Yes	0%
What are the potential psychological or spiritual applications of mescaline (could select more than one)	
Personal growth	90%
Spiritual growth	87%
Psychotherapeutic work	81%
Enhancing cognitive abilities	61%
Enhancing creative abilities	76%
Number of other people during session (also using mescaline)	4.7 (1.1)
Number of other people during session (not also using mescaline)	1.4 (5.6)

Variables may not be equal to 100% due to the rounding error. *N* is varied due to participants choosing “prefer not to answer” on specific items.

Regarding abuse potential, most respondents (55%) indicated that they never consumed more than one dose of mescaline in a session, and approximately one-third (32%) reported that they have consumed mescaline with other substances on one or two occasions. Additionally, very few respondents reported craving for mescaline (9%), ever being arrested or in legal trouble due to mescaline use (1%), or ever being in therapy or psychiatric treatment (<1%), and none reported seeking medical attention (0%), as a result of mescaline use. Moreover, most respondents (90%) reported that their mescaline use in the past year had decreased or remained the same, and almost all (97%) reported that they never attempted to quit using mescaline or to reduce their use.

### Characteristics of the “most memorable” mescaline experience

Overall, most respondents reported having the most memorable experience with San Pedro (48%; sample size, *N* = 218), and almost one-quarter (22%; *N* = 98) reported having the most memorable experience with Peyote. The remainder of the sample reported that they had the most memorable experience with synthetic (15%; *N* = 70) or extracted (15%; *N* = 66) mescaline. As shown in Table 3, most respondents across all four mescaline subgroups reported that their most memorable mescaline experience was a moderately high to very high dose, that it lasted between 8 and 13 h or longer, and that their main reason for consuming mescaline was for exploration (e.g. spiritual or personal) and connection (e.g. to self/nature). Overall, the sample reported using mescaline an average of three times prior, and four times since this most memorable mescaline experience. Most respondents (72%) reported consuming the mescaline without oversight of another person (i.e. self-administering the mescaline), and a smaller proportion reported purchasing their mescaline (37%) or growing or finding their mescaline (21%). In terms of the location of this most memorable mescaline experience, the largest proportion of respondents (41%) reported using mescaline outdoors.

**Table 3.** Characteristics of the “most memorable” mescaline experience in the full sample and comparisons of characteristics across mescaline subgroups.

Characteristic/variable	Total sample	Synthetic	San Pedro	Peyote	Extracted	χ^2^ or *F*	Post-hoc	Effect size
	*N* = 452	*N* = 70	*N* = 218	*N* = 98	*N* = 66			
	M (SD) or %	M (SD) or %	M (SD) or %	M (SD) or %	M (SD) or %			
Subjective dose						5.113	NS	NS
Low	7%	6%	6%	8%	11%			
Moderate	33%	31%	34%	32%	32%			
Moderately high	33%	36%	31%	32%	35%			
High	18%	16%	20%	16%	15%			
Very high	10%	11%	8%	12%	8%			
Number of doses (*n* = 450)	1.9 (1.8)	1.5 (1.3)	1.6 (1.4)	2.9 (2.5)	1.6 (1.9)	13.525**	P > S = SP = E	0.08
Duration of experience						16.995*		0.19
Less than 7 h	21%	26%	16%	32%	17%		P > SP	
8–10 h	24%	31%	23%	20%	24%			
11–13 h	26%	21%	30%	19%	26%			
More than 13 h	29%	21%	30%	29%	33%			
Number of previous mescaline experiences (*n* = 451)	3.2 (5.3)	2.6 (4.4)	2.9 (5.0)	4.2 (6.2)	3.4 (5.8)	1.645	NS	NS
Number of mescaline Experiences since (*n* = 451)	4.2 (6.5)	3.0 (5.1)	3.9 (6.1)	5.5 (7.9)	4.3 (6.7)	2.356	NS	NS
Location						24.363**		0.23
Indoors	34%	39%	38%	18%	42%		P < S = SP = E	
Outdoors	41%	43%	42%	40%	36%		NS	
Other (e.g. festival)	25%	19%	21%	42%	21%		P > S = SP = E	
Source of mescaline						122.398**		0.52
Purchased	37%	69%	34%	19%	41%		SP = P < E < S	
Gifted	11%	16%	6%	17%	14%		SP < S = P	
Grew/Found	21%	0%	30%	10%	30%		S < P < SP = E	
Provided (e.g. ceremony)	24%	1%	28%	43%	5%		E = S < SP < P	
Other	8%	14%	4%	10%	11%		S > SP	
Who administered the mescaline						46.246**		0.32
Self-administration	72%	93%	67%	57%	88%		S = E > SP = P	
Shamanic practitioner	20%	1%	27%	31%	3%		S = E < SP = P	
Other (e.g. peer and friend)	8%	6%	6%	12%	9%		NS	
Main reason for consuming mescaline (*n* = 451)						7.422	NS	NS
Explore/connect	81%	80%	83%	81%	77%			
Treatment/healing	7%	4%	9%	6%	5%			
Other (e.g. recreation)	12%	16%	9%	12%	18%			
Intensity of acute effects								
Mystical-type effects	3.2 (1.1)	3.1 (1.1)	3.2 (1.1)	3.3 (1.0)	3.2 (1.1)	0.247	NS	NS
Challenging effects	0.6 (0.6)	0.6 (0.6)	0.7 (0.7)	0.6 (0.5)	0.6 (0.5)	0.924	NS	NS
Psychological insight effects	2.4 (1.1)	2.1 (1.2)	2.4 (1.1)	2.5 (1.0)	2.2 (1.2)	2.085	NS	NS
Ego-dissolution effects	2.4 (1.3)	2.3 (1.3)	2.4 (1.3)	2.4 (1.3)	2.4 (1.3)	0.202	NS	NS
Personally meaningful	4.6 (1.4)	4.6 (1.4)	4.6 (1.4)	4.8 (1.3)	4.1 (1.6)	2.810*	P > E	.02
Spiritually significant	4.4 (1.8)	4.3 (1.9)	4.4 (1.8)	4.7 (1.7)	4.1 (2.0)	1.436	NS	NS
Psychologically challenging	2.8 (2.0)	2.5 (2.2)	2.7 (2.0)	3.1 (2.0)	2.8 (2.0)	1.629	NS	NS
Psychologically insightful	4.1 (7.8)	4.2 (1.8)	4.1 (1.8)	4.4 (1.6)	3.8 (1.9)	1.484	NS	NS
Enduring effects (changes in)								
Well-being/life satisfaction	2.1 (1.0)	2.1 (0.9)	2.1 (1.0)	2.1 (1.0)	1.8 (1.1)	1.792	NS	NS
Life’s purpose	1.8 (1.1)	1.6 (1.2)	1.8 (1.1)	2.0 (1.1)	1.6 (1.2)	2.546	NS	NS
Life’s meaning	1.8 (1.1)	1.7 (1.1)	1.8 (1.1)	2.0 (1.1)	1.5 (1.1)	2.334	NS	NS
Social relationships	1.6 (1.2)	1.5 (1.2)	1.6 (1.2)	1.7 (1.3)	1.4 (1.1)	0.687	NS	NS
Attitudes about life	1.9 (1.1)	1.9 (1.0)	1.9 (1.0)	2.0 (1.1)	1.7 (1.1)	1.337	NS	NS
Attitudes about self	1.8 (1.1)	1.6 (1.0)	1.8 (1.1)	1.9 (1.1)	1.7 (1.2)	1.234	NS	NS
Relationship to nature	2.0 (1.1)	1.9 (1.0)	2.0 (1.1)	2.2 (1.1)	1.9 (1.1)	1.296	NS	NS
Behaviors	1.5 (1.1)	1.4 (1.1)	1.6 (1.1)	1.5 (1.1)	1.9 (1.1)	0.669	NS	NS
How spiritual you are	1.6 (1.2)	1.6 (1.2)	1.6 (1.2)	1.9 (1.2)	1.3 (1.1)	2.870*	P > E	0.02
Attitudes about death	1.2 (1.2)	1.1 (1.2)	1.2 (1.2)	1.3 (1.3)	1.2 (1.2)	0.498	NS	NS
Views regarding the true nature of reality and the universe	1.7 (1.2)	1.7 (1.1)	1.7 (1.2)	1.9 (1.2)	1.3 (1.3)	3.089*	P > E	0.02
Mental health conditions before mescaline experience (Yes)								
Depression	44%	40%	47%	40%	44%	1.522	NS	NS
Anxiety	49%	49%	54%	39%	52%	5.521	NS	NS
Post-traumatic stress disorder	17%	13%	16%	18%	22%	2.011	NS	NS
Alcohol misuse/AUD	17%	22%	14%	19%	18%	3.117	NS	NS
Drug misuse/DUD	20%	24%	17%	18%	28%	4.498	NS	NS
Mental health conditions improve after experience (vs worsen/stay the same)^a^								
Depression	86%	89%	87%	83%	86%	0.449	NS	NS
Anxiety	80%	78%	83%	75%	76%	1.399	NS	NS
Post-traumatic stress disorder	76%	78%	75%	88%	64%	2.504	NS	NS
Alcohol misuse/AUD	67%	67%	66%	77%	55%	1.480	NS	NS
Drug misuse/DUD	68%	81%	60%	82%	59%	4.603	NS	NS

Variables may not equal 100% due to rounding error. *N* is varied due to participants choosing “prefer not to answer” on specific items. Scores of the mystical-type, challenging, insight, and ego-dissolution effects can range from 0 to 5. Ratings of personal meaning, spiritual significance, psychological challenge, and psychological insight can range from 0 to 7. Ratings of enduring effects can range from −3 to +3.

AUD: alcohol use disorder; DUD: drug use disorder; E: extracted; NS: non-significant; P: Peyote; S: synthetic; SP: San Pedro.

a Among those who reported having the psychiatric condition.

* *p* < 0.05. **p < 0.001.

As shown in Table 3, there were several differences across the four mescaline subgroups, wherein those in the Peyote subgroup reported consuming more doses (2.9) in their most memorable experience compared to all other subgroups. Additionally, a larger proportion of respondents in the Peyote subgroup (32%) reported a total duration of the experience less than 7 h compared to those in the San Pedro subgroup. There was also a smaller proportion of respondents in the Peyote subgroup (18%) who reported using mescaline indoors compared to all other subgroups. Sources of mescaline also varied by subgroup, in that there was a larger proportion of respondents in the synthetic subgroup (69%) reporting purchasing their mescaline, a smaller proportion of those in the San Pedro subgroup reported (6%) having been gifted mescaline, and a larger proportion of those in the Peyote subgroup reported (43%) that their mescaline was provided in a ceremony, compared to all other subgroups in each of those comparisons. Likewise, larger proportions of respondents in the San Pedro (27%) and Peyote (31%) subgroups reported that their mescaline was administered by a shamanic practitioner compared to those in the synthetic or extracted subgroups.

### Subjective effects of the “most memorable” mescaline experience

The intensity of acute subjective mescaline effects was examined across the sample and within each mescaline subgroup. Overall, respondents rated acute mystical-type effects as “moderate,” challenging effects as “very slight,” psychological insight effects as “slight” and ego-dissolution effects as “slight.” These averages should be considered in light of a range of doses reportedly consumed in this sample (e.g. 40% of the sample reported consuming a “low” to “moderate” dose of mescaline in this most memorable experience), which could explain these average ratings. There were no significant differences in the ratings of the intensity of these acute subjective effects as a function of a mescaline subgroup. See Table 3 for details.

Regarding the enduring effects associated with ones most memorable mescaline experience, approximately one-quarter to one-third (rates varied from 28% to 35%) of the sample rated their mescaline experience as one of the top five or single most personally meaningful, spiritually significant, or psychologically insightful experiences of their entire lives. A smaller proportion of respondents (11%) rated the experience as one of the top five or single most psychologically challenging. There were no significant differences in these proportions as a function of a mescaline subgroup.

### Effect of the “most memorable” mescaline experience on psychological functioning

Almost half of respondents reported that prior to the most memorable mescaline experience, they had anxiety (49%) or depression (44%), with smaller proportions reporting a history of drug misuse or use disorder (20%), post-traumatic stress disorder (17%), and alcohol misuse or use disorder (17%) (see Table 3). There were no significant differences in the rates of these mental health conditions as a function of a mescaline subgroup. Furthermore, of the respondents who reported having a prior psychiatric condition, large proportions reported that the condition improved (depression better = 86%; anxiety better = 80%; post-traumatic stress disorder better = 76%; drug misuse or use disorder better = 68%; and alcohol misuse or use disorder better = 67%) following their most memorable mescaline experience. Similarly, there were no significant differences in the rates of improvement of these mental health conditions as a function of a mescaline subgroup.

## Discussion

To the best of our knowledge, this is the first international epidemiological study on mescaline use. The present data indicate that most people infrequently used San Pedro or Peyote orally through self-administration (i.e. consuming the mescaline without oversight of another person) for spiritual and nature connection. The infrequent pattern of use is similar to findings in a former survey-based study on 5-MeO-DMT (5-methoxy-N,N-dimethyltryptamine; Davis et al., 2018) and echoes previous knowledge, suggesting the low abuse potential of psychedelics (Johansen and Krebs, 2015; Krebs and Johansen, 2013). The motivation for mescaline use is similar to reports from other research on psilocybin and ayahuasca used in a naturalistic setting (Mason et al., 2019; Uthaug et al., 2018b), as well as the previous survey study on 5-MeO-DMT, where respondents reported using 5-MeO-DMT for the purpose of spiritual exploration (Davis et al., 2018).

The respondents’ ratings (very slight to moderate) of the various subjective effects (mystical, ego-dissolution, psychological insight, and challenge) are inconsistent with that of other psychedelics (ayahuasca and psilocybin) (Griffiths et al., 2011; MacLean et al., 2011; Uthaug et al., 2018a) and could be attributed to mescaline’s low potency (Dasgupta, 2019) and the reported dose (low to moderate) by 40% of the respondents in this sample. Additionally, very few respondents in the present study reported legal problems, psychological difficulties, or craving of mescaline, and none reported medical difficulties. These numbers are similar to reports from 5-MeO-DMT users (Davis et al., 2018) and relatively low in comparison to reported craving for more widely used substances (e.g. alcohol) (McCabe et al., 2017). Consistent with prior research (Johansen and Krebs, 2015; Krebs and Johansen, 2013), these findings may indicate that mescaline has a relatively favorable psychological safety profile for the use in naturalistic settings as evidenced by reports of low abuse liability. However, it is possible that people who have had negative experiences with mescaline might have been less likely to have seen or responded to the present survey, which could have biased our findings. Additionally, definitive safety profiles that include the assessments of vital signs, blood pressure, and electrocardiography (ECG) need to be established in laboratory studies of mescaline administration.

Most respondents with prior psychiatric conditions (i.e. depression, anxiety, post-traumatic stress disorder, and drug and alcohol misuse) reported improvements in these conditions following their most memorable experience with mescaline. One can speculate whether the experience was memorable due to the improvement in such health functioning. Nevertheless, this result is consistent with previous findings from observational and clinical research that demonstrated subjective and objective improvements in mental health-related variables, following the use of psychedelics (Carhart-Harris et al., 2016; Davis et al., 2019, 2020b; Palhano-Fontes et al., 2015; Roseman et al., 2018; Uthaug et al., 2018a, 2019a, 2019b), including previous knowledge about the potential for psychedelics to help treat addiction (Garcia-Romeu et al., 2019, 2020; Johnson et al., 2014; Talin and Sanabria, 2017; Winkelman, 2014). It is plausible that several factors (e.g. set and setting, acute drug effects, and neurological changes; Clarke et al., 2013; Fox et al., 2017) moderate the outcome of psychedelic experiences on positive mental health and substance use outcomes. Nevertheless, future controlled studies should explore the role of mechanisms of drug action for mescaline using rigorous controlled methods.

Results from this study also showed no significant differences in the subjective acute and enduring effects between mescaline types. Although this may indicate relatively minimal or no differences in the acute and enduring effects of different types of mescaline, rigorous controlled studies could reveal potential differences between them. While all groups exhibited broad similarities, the Peyote subgroup reported consuming more doses compared to other groups. This might be due to the bitter taste of Peyote, which is known to induce nausea and vomiting (Erowid, 2009; Nolte and Zumwalt, 1999). It is possible that participants in the Peyote subgroup experienced emesis that can have warranted repeated dosing to obtain desired subjective effects. Additionally, it is not clear why the Peyote subgroup reported shorter duration of effects, but one could speculate if it could be due to lower net intake of mescaline (despite increased number of doses ingested), due to the alkaloid ratio of the cacti, dose, and experience of emesis. Finally, both San Pedro and Peyote subgroups reported using it in a ceremonial context administered by a shamanic practitioner, which is consistent with previous knowledge about historical use of Peyote (Dasgupta, 2019).

This study is not without limitations. For example, the present survey aimed to assess a person’s “*most memorable experience with mescaline*”; however, we did not control for period of memory recall, attitudes toward mescaline, lifetime use of mescaline, and potential confounds associated with concurrent use of other compounds. Furthermore, the cross-sectional nature of the data precludes any interpretation of causality regarding the short-term and long-term effects of mescaline. Additionally, we used a different scoring scale of the EDI created by Nour et al. (2017), and the survey sample was recruited using Internet advertisements and was therefore subjected to selection bias (e.g. access to the Internet, willingness to participate, and cultural background). Moreover, as with other web-based studies of people using licit and illicit substances (Ashrafioun et al., 2016), the sample was included mostly of White heterosexual men, who could reflect a limitation in the recruitment method. Previous research and literature show that mescaline is used in many Spanish-speaking populations (e.g. Mexico and Peru) (Dobkin, 1968; Jay, 2019; Smythies, 1953); thus, it follows that future research should attempt to recruit samples including individuals who identify as being speakers of Spanish languages in order for the sample to faithfully reflect populations who tend to use mescaline.

It is also noteworthy that the research team made explicit attempts at reaching out to the NA population and other Indigenous communities internationally. However, <10 respondents completed surveys identified as being either NA or as Indigenous. Though a much larger survey sample would have been optimal, it is not surprising that a limited number of submissions were received due to historical trust issues that NA and Indigenous people carry regarding the impacts that Western scientific studies can have on these cultures. For NA people of the US, Peyote is and has been a sensitive and controversial topic. Peyote is revered by NA people for its medicinal qualities, but its reverence holds a deep connection with the people to the land and the environment. Due to these relationships, Western scientific studies that involve Peyote with NA and Indigenous people are limited. There is not only a reluctance to take part in Western studies with plants that are considered sacrilegious to the NA population but also hesitancy with plants like Peyote. Where Peyote is a foundational element of the NAC and a recognized religious right and ceremonial practice, the knowledge about the ceremony and ceremonial experiences is not easily shared with outsiders.

Finally, although we have compared mescaline experiences by mescaline type of use in the present study, we do not intend for these data to be interpreted to mean that further rigorous, clinical research are not needed. We cannot conclude that similarities or differences observed in this dataset may have also been caused by a variety of additional factors, such as participant demographics, “set and setting” (i.e. contextual variables) that might co-vary with the type of use. Therefore, the present observations should be replicated in controlled clinical trials to allow any strong conclusion.

## Conclusion

Despite the limitations described above, the present findings indicate that mescaline, in any form, may produce a psychedelic experience that is associated with meaningful and spiritually significant experiences, improvements in mental health, and has low probability for increased use and misuse. Further research among NA and other Indigenous populations could provide further understanding of their historical and long-term ceremonial use. Finally, future examination in naturalistic and clinical settings are also needed to confirm and expand on the current findings of the overall study.
